# AutismKB 2.0: a knowledgebase for the genetic evidence of autism spectrum disorder

**DOI:** 10.1093/database/bay106

**Published:** 2018-10-18

**Authors:** Changhong Yang, Jiarui Li, Qixi Wu, Xiaoxu Yang, August Yue Huang, Jie Zhang, Adam Yongxin Ye, Yanmei Dou, Linlin Yan, Wei-zhen Zhou, Lei Kong, Meng Wang, Chen Ai, Dechang Yang, Liping Wei

**Affiliations:** 1 College of Life Sciences, Beijing Normal University, Beijing, China; 2 National Institute of Biological Sciences, Beijing, China; 3 Institute of Infectious Diseases, Beijing Key Laboratory of Emerging Infectious Diseases, Beijing Ditan Hospital Capital Medical University, Beijing, China; 4 Peking-Tsinghua Center for Life Sciences, Beijing, China; 5 School of Life Sciences, Peking University, Beijing, China; 6 Center for Bioinformatics, State Key Laboratory of Protein and Plant Gene Research, School of Life Sciences, Peking University, Beijing, China; 7 Academy for Advanced Interdisciplinary Studies, Peking University, Beijing, China; 8 State Key Laboratory of Cardiovascular Disease, Beijing Key Laboratory for Molecular Diagnostics of Cardiovascular Diseases, Diagnostic Laboratory Service, Fuwai Hospital, National Center for Cardiovascular Diseases, Chinese Academy of Medical Sciences and Peking Union Medical College, Beijing, China

## Abstract

Autism spectrum disorder (ASD) is a complex neurodevelopmental disorder with strong genetic contributions. To provide a comprehensive resource for the genetic evidence of ASD, we have updated the Autism KnowledgeBase (AutismKB) to version 2.0. AutismKB 2.0 integrates multiscale genetic data on 1379 genes, 5420 copy number variations and structural variations, 11 669 single-nucleotide variations or small insertions/deletions (SNVs/indels) and 172 linkage regions. In particular, AutismKB 2.0 highlights 5669 *de novo* SNVs/indels due to their significant contribution to ASD genetics and includes 789 mosaic variants due to their recently discovered contributions to ASD pathogenesis. The genes and variants are annotated extensively with genetic evidence and clinical evidence. To help users fully understand the functional consequences of SNVs and small indels, we provided comprehensive predictions of pathogenicity with iFish, SIFT, Polyphen etc. To improve user experiences, the new version incorporates multiple query methods, including simple query, advanced query and batch query. It also functionally integrates two analytical tools to help users perform downstream analyses, including a gene ranking tool and an enrichment analysis tool, KOBAS. AutismKB 2.0 is freely available and can be a valuable resource for researchers.

## Introduction 

Autism spectrum disorder (ASD) is a severe neurodevelopmental disorder with core symptoms that include deficits in social interaction and social communication, as well as stereotypical and repetitive behaviors (1). Epidemiological studies in many countries have shown that the prevalence of ASD ranges from 1 to 2% of the population (2, 3). Twin studies and cohort studies have established that genetic factors play a major role in the etiology of ASD (4–6). Inherited mutations and *de novo* mutations have both been found to contribute significantly to ASD (7–15). More recently, postzygotic genomic mosaicisms have also been associated with ASD (16–18).

Because of a highly heterogeneous genetic etiology, thousands of genes have been reported to be associated with ASD (19). These genes were identified with a variety of experimental approaches with variable evidence over a long period of time by many different groups. Thus, there is a strong need for databases that collect comprehensive evidence about ASD-associated genes from the extensive literature and research information resources. Autism KnowledgeBase (AutismKB), developed by our group in 2011, was the largest such database; its initial release included 2193 ASD genes, 2806 single nucleotide polymorphisms (SNPs) and indels, 4544 copy number variations (CNVs) and structural variations (SVs) and 158 linkage regions (20). Three other autism-related genetic databases are available to researchers. The Autism Chromosome Rearrangement Database (21) includes 372 ASD-associated chromosomal breakpoints, whereas the Autism Genetic Database (22) includes 743 CNVs of 226 ASD genes, and the AutDB (23) includes 2225 CNVs of 990 genes and 1165 animal models.

Since its publication, AutismKB has received 1 533 725 page views from 42 619 unique Internet Protocol (IP) addresses. However, new research developments, especially those fueled by next-generation sequencing (NGS) technologies, have revealed many new ASD-related genes and genetic variants, as well as new types of genetic variation, such as *de novo* variants and mosaic variants (16–18). Large-scale NGS studies revealed that *de novo* variants have important contributions to ASD (7, 9, 10, 14, 24–26) and might explain >10% of ASD probands (27). Dou *et al.* (17) estimated that 2.6% of the ASD diagnoses in the Simons Simplex Collection (SSC) could be explained by mosaic variants arising postzygotically in probands.

Here, in an effort to help researchers keep pace with the rapid growth in ASD-related genetic information, we updated AutismKB to version 2.0 (http://db.cbi.pku.edu.cn/autismkb_v2/) with significant expansion and changes.

## Materials and methods

### The framework of AutismKB 2.0 

**Figure 1:**
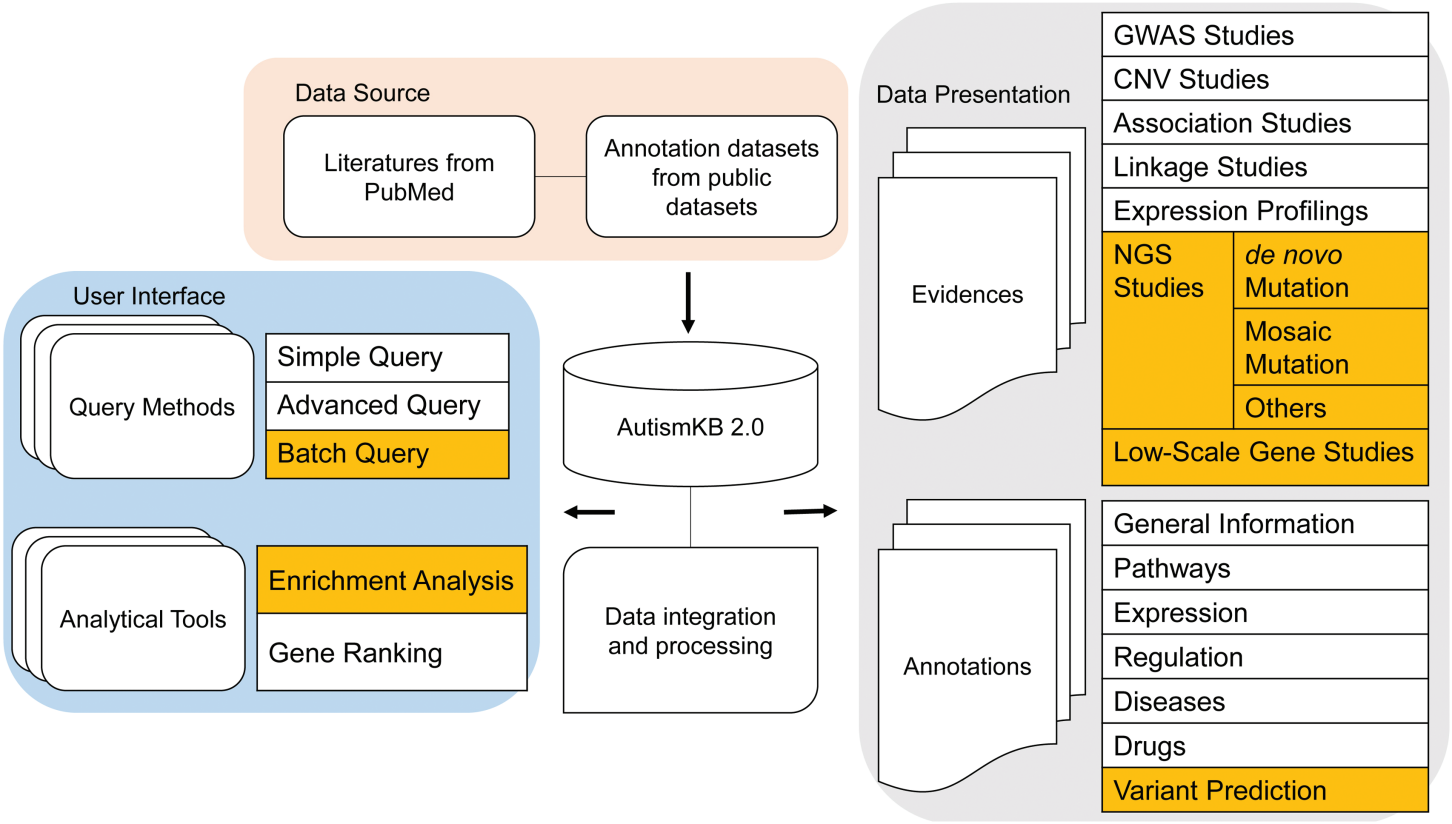
Overview of the structure of AutismKB 2.0. Newly added or modified modules are highlighted.

AutismKB 2.0 was created as a relational database using MySQL Server 5.6.26. The web interfaces were designed using PHP (5.5.18-pl0-gentoo), JavaScript and HTML. An overview of the construction of AutismKB 2.0 is shown in Figure 1. The framework consists of three major parts. The first part collects and updates autism-related genetic data and annotated data sets. The second part archives and presents the nine evidence data types and seven annotation data types. The third part is the user interface that displays our main data sets, three query methods and two analytical tools on our website. In this new version, we added new content and made corresponding changes to these three parts. In the first part, we added a new collection of mosaic-related literature. In the second part, we added mosaic variants as a new data type, as well as variant prediction in the annotations. In the user interface, we added new tools for batch query and enrichment analysis. We also changed the categories of data by adding the category of *de novo* and mosaic variants, introducing function predictions, collecting large-scale single-nucleotide variants (SNVs) in the categories of NGS and optimizing the data table structure and table contents in the back end to accelerate the access speed and to elevate the user experience.

### Data collection 

We conducted a systemic review of the ASD-related literature by using the query term `autis^*^[Title/Abstract]’ to search the PubMed database monthly, and we updated the database every 6 months. For mosaic mutations, we used the query term `autis^*^ and mosaic^*^’ to search the PubMed database. Next, we manually reviewed the search results. We collected genes, variations and evidence from the literature and integrated them into AutismKB 2.0. The selection criterion for the literature is as follows: defined ASD-related genes were presented. For all publications that met this requirement, a double recheck for ASD genetic information in the literature was carried out.

All genes and variants reported in the literature were divided into nine categories based on the primary experimental methodologies of the studies in which they were reported, including `Genome-Wide Association Studies (GWAS)’, `Genome-Wide Copy Number Variation/Structure Variation (CNV/SV) Studies’, `Linkage Studies’, `Low-Scale Genetic Association Studies’, `Expression Profilings’, `NGS *de novo* Mutation Studies’, `NGS Mosaic Mutation Studies’, `NGS Other Studies’ and `Low-Scale Gene Studies’.

### Functional annotations 

To better demonstrate the functional aspects of ASD-related genes, extensive information, including their nucleotide and protein sequences, gene ontology (GO), expression profiles among tissues, regulatory information and pathway and disease-related information, was retrieved from online database, which included NCBI gene, NCBI GEO, NCBI Unigene, GO, OMIM, HGNC, Ensembl, Uniprot, BioGRID, BIND, HPRD, AlzGene, PDGene, SZGene, MGI, ZFIN, FB, BioGPS, Allen Brain Atlas, PRIDE, Peptide Atlas, dbPTM, miRWalk, Tarbase, NATs, CTD, PharmGKB and DrugBank.

To provide evidence of the functional consequences of the reported variants, we added the predicted pathogenicity of genetic variants based on ANNOVAR (28) with Refseq (build hg19) and iFish (integrated functional inference of SNVs in human) (29). iFish is a supporting vector machine-based classifier that uses gene-specific and gene family-specific attributes. At the same time, iFish provides functional annotations from other classifiers such as SIFT (30), Polyphen2 (31) and MutationTaster2 (32). iFish utilizes a customized prediction cut-off for each classifier that maximizes the sum of sensitivity and specificity.

To provide a user-tunable gene list with the strongest possible evidence, we provided a gene ranking algorithm identical to that included in AutismKB 1.0 (20). Briefly, we used an evidence-based candidate gene prioritization approach (33) that first assigns different weights to different types of experimental evidence using a benchmark ASD gene set, after which it calculates the weight of evidence of each gene by summing the weights of the positive evidence for that gene.

**Table 1:** Raw scoring criteria and number of genes for each type of evidence

**Experimental methods**	**Raw score**	**Number of genes in AutismKB 2.0**	**Number of genes in AutismKB**
GWAS	Score 1: one positive study (*P* ≤ 1e-5)	176	81
	Score 2: two positive studies and *P* > 1e-7	31	46
	Score 3: two positive studies and *P* ≤ 1e-7	5	5
CNV/SV studies	Score 1: 1–3 positive studies	151	128
	Score 2: 4–8 positive studies	36	23
	Score 3: ≥9 positive studies	19	12
Linkage analyses	Score 1: 1–3 positive studies	5052	535
	Score 2: 4–8 positive studies	183	43
	Score 3: ≥9 positive studies	0	0
Low-scale genetic association studies	Score 1: one positive study (*P* ≤ 0.05)	4413	1086
	Score 2: two or more positive studies and *P* > 0.001	321	34
	Score 3: two or more positive studies and *P* ≤ 0.001	18	19
Expression profilings	Score 1: one positive study	1335	1320
	Score 2: two positive studies	291	285
	Score 3: three or more positive studies	62	50
NGS *de novo* mutation studies	Score 1: one positive study	635	
	Score 2: two positive studies	104	
	Score 3: three or more positive studies	18	
NGS mosaic mutation studies	Score 1: one positive study	116	
	Score 2: two positive studies	12	
	Score 3: three or more positive studies	2	
NGS other studies	Score 1: one positive study	116	
	Score 2: two positive studies	12	
	Score 3: three or more positive studies	2	
Low-scale gene studies	Score 1: one positive study	133	
	Score 2: two positive studies	17	
	Score 3: three or more positive studies	1	

### Improved scoring system for ranking ASD candidate genes 

AutismKB 2.0 implemented an improved gene scoring algorithm compared to AutismKB 1.0. First, we extended the six categories of experimental evidences to nine categories by dividing the previous category `NGS and Low-Scale Gene Studies’ into four different categories including `NGS *de novo* Mutation Studies’, `NGS Mosaic Mutation Studies’, `NGS Other Studies’ and `Low-Scale Gene Studies’. For missense mutations identified from NGS studies, we only considered those predicted `deleterious’ by iFish as supportive evidence for ASD pathogenesis. The criteria and statistics of raw scores for each type of evidence are shown in Table 1. Second, we updated the benchmark data set. In AutismKB 1.0, the benchmark data set was comprised of 21 non-syndromic autism-related genes from six review papers published before 2010 (34–39). On comparison, AutismKB 2.0 used a more up-to-date benchmark data set consisting of 46 non-syndromic autism-related genes recommended by Simons Foundation Powering Autism Research for Knowledge (40). Third, the range of weights for each evidence type in AutismKB 2.0 was changed from 1–7 to 1–10, and the number of possible weight combination was dramatically increased from 7^6^ to 10^9^. Fourth, we re-benchmarked and updated the optimal weight matrix recommended by AutismKB 2.0, by ranking the 75th percentile of the benchmark data set to the highest rank (Supplementary Table 2). The AutismKB 2.0 web server also allows users to choose their own weights freely for each type of experimental evidence, as well as the cutoffs.

To help users perform downstream analyses, we integrated an enrichment analysis tool, KOBAS (41), into the new version. After a user uploads a list with gene symbols, AutismKB 2.0 automatically searches the background database. If the target genes are present in the database, the server will automatically convert their symbols to the appropriate Entrez gene indexes. Next, the website automatically submits the list to KOBAS for enrichment analysis. Finally, users can view and download the enriched functional categories from their queried gene lists.

### Update plan for AutismKB 2.0

To keep AutismKB 2.0 up-to-date in the future, we plan to collect ASD-related literatures from PubMed every month, which will be classified into nine categories according to their experimental methods. We will then extract the phenotype and genotype data from each study. The collected data will be manually curated every 6 months and uploaded to the back-end database through a Perl-based script. We will also recalibrate the scoring system of each evidence and gene and post an update log on the AutismKB 2.0 website (http://db.cbi.pku.edu.cn/autismkb_v2/new.php).

## Results and discussion

### Database summary

We reviewed the abstracts of 13 749 published studies up to 30 June 2018 and retrieved the full text of 3208 selected studies. If the abstract of the literature provided phenotype and genotype information that fulfilled our requirements, the information was extracted directly from the abstract; otherwise, the genotype and phenotype information was extracted from the main text and/or the supplementary materials. With the rapid increase in the amount of data from NGS and other related studies, we have increased the amount of literature from NGS, and especially *de novo* mutation studies and mosaic mutation studies. Information from NGS studies was included in the sixth kind of evidence in the database.

We updated the knowledgebase every 6 months as shown in Supplementary Figure 1. Since the initial release of AutismKB in 2012, 1036 new research articles were added into AutismKB 2.0, including 22 GWAS studies, 230 CNV/SV studies, 26 linkage studies, 338 association studies, 15 expression studies, 43 NGS *de novo* mutation studies, 6 NGS mosaic mutation studies, 37 NGS other studies and 319 low-scale gene studies. In summary, AutismKB 2.0 currently includes 5420 CNVs/SVs, 5669 *de novo* mutations, 789 mosaic mutations and 172 linkage regions.

Recent studies have shown that postzygotic mosaic mutations are an important, yet underestimated, genetic risk factor for ASD (16–18, 42–47). AutismKB 2.0 is the only ASD database to include germline variants, 789 mosaic SNV, and 6 mosaic CNVs, including 583 mosaic variants detected and validated in the whole exome sequencing data of 5947 families collected by SSC and Autism Sequencing Consortium, as well as 247 unvalidated, yet highly confident, mosaic mutations from the sequencing data of 2264 families.

To conclude, compared with the initial version of AutismKB, the number of articles for GWAS, CNV, linkage, association, expression, NGS and other studies increased by 144, 165, 18, 57, 25 and 74%, respectively, in AutismKB 2.0 (Table 2), and mosaic mutations were included as an independent evidence type in the new version for the first time.

**Table 2:** The comparison of data collected in AutismKB and AutismKB 2.0

**Evidence Type**	**AutismKB**	**AutismKB 2.0**
GWAS	9	22
CNV/SV studies	85	230
Linkage analyses	22	26
Low-scale genetic association studies	215	338
Expression profilings	12	15
NGS *de novo* mutation studies		43
NGS mosaic mutation studies	236	6
NGS other studies		37
Low-scale gene studies		319
Total	579	1036

**Figure 2:**
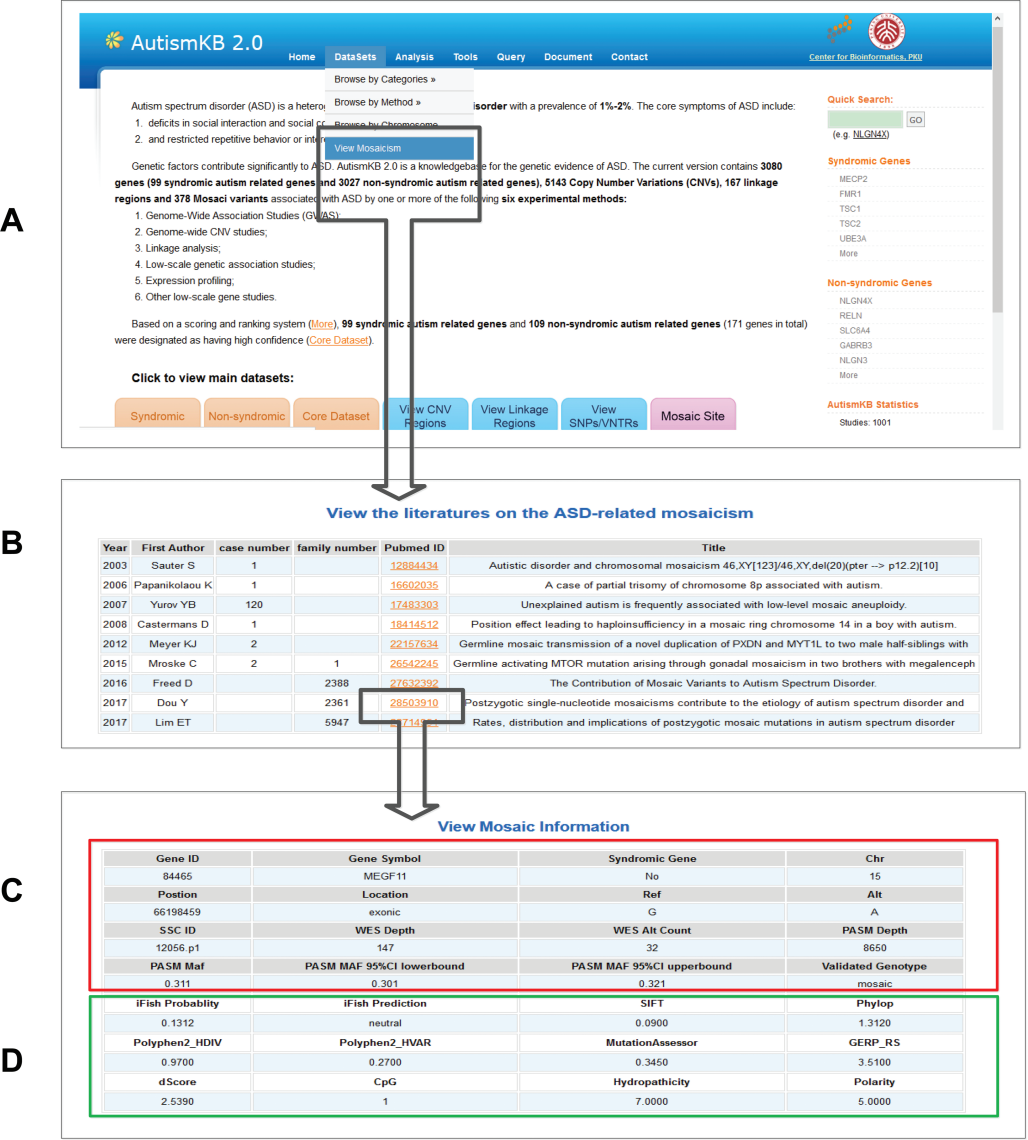
Examples of the webpages of AutismKB 2.0. (A) The data set link for mosaic mutations. (B) List of studies related to ASD-related mosaicism. **(C and D)** Detailed general information (C) and functional prediction (D) of a mosaic mutation in *RELN*.

### Database interface and access

In the updated version 2.0 of AutismKB, we improved the user interface by adding `variant’, `View Mosaicism’, `enrichment analysis’ and `batch query’ entrances (Figure 1). Among these, the `variant’ entrances included CNV/SVs, SNVs/indels, mosaics and linkage regions previously provided under CNV, linkage, NGS and other categories.

To accelerate the user navigation speed and improve the user experience, we optimized the database by adding tables containing mosaic information, tables containing functional annotations, tables with updated polymorphism information such as dbsnp150 and other information. The dbsnp150 table replaced the out-of-date table snp130. We also changed the table structure of gene_score and all_variants. The database now includes ∼91 different tables. Tables now include keys such as PubMed id, Entrez id, SNV id, Mosaic id, iFish id, CNV id and linkage id, which serve as the index between all tables.

### Update of the gene annotation

We annotated the ASD-related genes with extensive information, including gene name and id, sequence, functional annotation, animal models, expression, regulation, pathways, associated diseases and related drugs. These annotations can help users to understand more information about these genes. Additionally, we have now added predicted pathogenicity sores and annotation about ASD-related gene variants (Figure 2 and Supplementary Table 1). A total of 6672 SNV were included in AutismKB 2.0. Among 3615 exonic missense variants, 1718 (47.5%) were predicted to be pathogenic by iFish, whereas 1897 (52.5%) were predicted to be neutral. This information may help users evaluate and rank genetic variants in their research.

### Conclusion and future perspective

ASD is not a Mendelian disease. Rather, it is a complex and highly heterogeneous disease. Thousands of genes have been reported to be associated with ASD (10–13, 48, 49). To provide a comprehensive and useful knowledgebase, we have updated AutismKB to version 2.0. We used the gene scoring algorithm and the latest benchmark data set to rank the genes collected in the database. In addition to 99 syndromic genes, we selected 1280 non-syndromic genes with a total score greater than four as candidate ASD-associated genes (Supplementary Table 3). Among them, 30 syndromic and 198 non-syndromic genes with a total score greater than 16 were designated as high-confidence ASD-associated genes (Supplementary Table 4).

We will continue to maintain and update AutismKB 2.0 in the future, so that it will provide increased utility to the community. We plan to continue to read and integrate the ASD-related literature to collect data for ASD genes. One limitation of the database is that it does not contain detailed phenotypic information related to ASD genes. Therefore, we plan to follow up with the latest research methods to integrate ever more helpful annotations for ASD genes, including phenotypic scores for ASD probands. For example, if the literature reports Autism Diagnostic Interview Review (ADI-R) and/or Autism Diagnostic Observation Schedule (ADOS) scores, we will collect the detailed scores, which are strongly correlated with the severity of ASD symptoms. Another potential resource of phenotypic data is from public databases such as the Human Phenotype Ontology (HPO) (50). In the future, we plan to extract the ASD-related gene and phenotype information from HPO and integrate them into AutismKB 2.0.

In summary, AutismKB 2.0 integrates multiscale evidence and detailed genetic information for ASD-related genes. We believe that this updated database will greatly facilitate ongoing and future research about ASD.

## Supplementary Material

Supplementary Data

## Notes

**Database URL**: http://db.cbi.pku.edu.cn/autismkb_v2

## References

[1] American Psychiatric Association (2013) *DSM 5*. American Psychiatric Publishing, Arlington, VA, USA.

[2] Blumberg S.J., Bramlett M.D., Kogan M.D. et al. (2013) Changes in prevalence of parent-reported autism spectrum disorder in school-aged U. S. children: 2007 to 2011–2012. *Natl. Health Stat. Report.*, 65, 1–11. 24988818

[3] Christensen D.L., Baio J., Van Naarden Braun K. et al. (2016) Prevalence and characteristics of autism spectrum disorder among children aged 8 years—autism and developmental disabilities monitoring network, 11 sites, United States, 2012. *MMWR Surveill. Summ.*, 65, 1–23. 10.15585/mmwr.ss6503a1 PMC7909709 27031587

[4] Hallmayer J., Cleveland S., Torres A. et al. (2011) Genetic heritability and shared environmental factors among twin pairs with autism. *Arch. Gen. Psychiatry*, 68, 1095. 21727249 10.1001/archgenpsychiatry.2011.76 PMC4440679

[5] Klei L., Sanders S.J., Murtha M.T. et al. (2012) Common genetic variants, acting additively, are a major source of risk for autism. *Mol. Autism*, 3, 9. 23067556 10.1186/2040-2392-3-9 PMC3579743

[6] Gaugler T., Klei L., Sanders S.J. et al. (2014) Most genetic risk for autism resides with common variation. *Nat. Genet.*, 46, 881–885. 25038753 10.1038/ng.3039 PMC4137411

[7] Dong S., Walker M.F., Carriero N.J. et al. (2014) De novo insertions and deletions of predominantly paternal origin are associated with autism spectrum disorder. *Cell Rep.*, 9, 16–23. 25284784 10.1016/j.celrep.2014.08.068 PMC4194132

[8] Sanders S.J., He X., Willsey A.J. et al. (2015) Insights into autism spectrum disorder genomic architecture and biology from 71 risk loci. *Neuron*, 87, 1215–1233. 26402605 10.1016/j.neuron.2015.09.016 PMC4624267

[9] Iossifov I., O’Roak B.J., Sanders S.J. et al. (2014) The contribution of de novo coding mutations to autism spectrum disorder. *Nature*, 515, 216–221. 25363768 10.1038/nature13908 PMC4313871

[10] Sanders S.J., Murtha M.T., Gupta A.R. et al. (2012) De novo mutations revealed by whole-exome sequencing are strongly associated with autism. *Nature*, 485, 237–241. 22495306 10.1038/nature10945 PMC3667984

[11] Neale B.M., Kou Y., Liu L. et al. (2012) Patterns and rates of exonic de novo mutations in autism spectrum disorders. *Nature*, 485, 242–245. 22495311 10.1038/nature11011 PMC3613847

[12] O’Roak B.J., Vives L., Girirajan S. et al. (2012) Sporadic autism exomes reveal a highly interconnected protein network of de novo mutations. *Nature*, 485, 246–250. 22495309 10.1038/nature10989 PMC3350576

[13] Iossifov I., Ronemus M., Levy D. et al. (2012) De novo gene disruptions in children on the autistic spectrum. Neuron, 74, 285–299. 22542183 10.1016/j.neuron.2012.04.009 PMC3619976

[14] Michaelson J.J., Shi Y., Gujral M. et al. (2012) Whole-genome sequencing in autism identifies hot spots for de novo germline mutation. Cell, 151, 1431–1442. 23260136 10.1016/j.cell.2012.11.019 PMC3712641

[15] Jiang Y.H., Yuen R.K., Jin X. et al. (2013) Detection of clinically relevant genetic variants in autism spectrum disorder by whole-genome sequencing. *Am. J. Hum. Genet.*, 93, 249–263. 23849776 10.1016/j.ajhg.2013.06.012 PMC3738824

[16] Freed D. and Pevsner J. (2016) The contribution of mosaic variants to autism spectrum disorder. *PLoS Genet.*, 12, e1006245. 27632392 10.1371/journal.pgen.1006245 PMC5024993

[17] Dou Y., Yang X., Li Z. et al. (2017) Postzygotic single-nucleotide mosaicisms contribute to the etiology of autism spectrum disorder and autistic traits and the origin of mutations. *Hum. Mutat.*, 38, 1002–1013. 28503910 10.1002/humu.23255 PMC5518181

[18] Lim E.T., Uddin M., De Rubeis S. et al. (2017) Rates, distribution and implications of postzygotic mosaic mutations in autism spectrum disorder. *Nat. Neurosci.*, 20, 1217–1224. 28714951 10.1038/nn.4598 PMC5672813

[19] Huguet G., Ey E. and Bourgeron T. (2013) The genetic landscapes of autism spectrum disorders. *Annu. Rev. Genomics Hum. Genet.*, 14, 191–213. 23875794 10.1146/annurev-genom-091212-153431

[20] Xu L.M., Li J.R., Huang Y. et al. (2012) AutismKB: an evidence-based knowledgebase of autism genetics. *Nucleic Acids Res.*, 40, D1016–D1022. 22139918 10.1093/nar/gkr1145 PMC3245106

[21] Marshall C.R., Noor A., Vincent J.B. et al. (2008) Structural variation of chromosomes in autism spectrum disorder. *Am. J. Hum. Genet.*, 82, 477–488. 18252227 10.1016/j.ajhg.2007.12.009 PMC2426913

[22] Matuszek G. and Talebizadeh Z. (2009) Autism Genetic Database (AGD): a comprehensive database including autism susceptibility gene-CNVs integrated with known noncoding RNAs and fragile sites. *BMC Med. Genet.*, 10, 102. 19778453 10.1186/1471-2350-10-102 PMC2761880

[23] Basu S.N., Kollu R. and Banerjee-Basu S. (2009) AutDB: a gene reference resource for autism research. *Nucleic Acids Res.*, 37, D832–D836. 19015121 10.1093/nar/gkn835 PMC2686502

[24] O’Roak B.J., Deriziotis P., Lee C. et al. (2011) Exome sequencing in sporadic autism spectrum disorders identifies severe de novo mutations. *Nat. Genet.*, 43, 585–589. 21572417 10.1038/ng.835 PMC3115696

[25] Wang T., Guo H., Xiong B. et al. (2016) De novo genic mutations among a Chinese autism spectrum disorder cohort. *Nat. Commun.*, 7, 13316. 27824329 10.1038/ncomms13316 PMC5105161

[26] Turner T.N., Yi Q., Krumm N. et al. (2017) denovo-db: a compendium of human de novo variants. *Nucleic Acids Res.*, 45, D804–D811. 27907889 10.1093/nar/gkw865 PMC5210614

[27] Ronemus M., Iossifov I., Levy D. et al. (2014) The role of de novo mutations in the genetics of autism spectrum disorders. *Nat. Rev. Genet.*, 15, 133–141. 24430941 10.1038/nrg3585

[28] Wang K., Li M. and Hakonarson H. (2010) ANNOVAR: functional annotation of genetic variants from high-throughput sequencing data. *Nucleic Acids Res.*, 38, e164. 20601685 10.1093/nar/gkq603 PMC2938201

[29] Wang M. and Wei L. (2016) iFish: predicting the pathogenicity of human nonsynonymous variants using gene-specific/family-specific attributes and classifiers. *Sci. Rep.*, 6, 31321. 27527004 10.1038/srep31321 PMC4985647

[30] Ng P.C. and Henikoff S. (2001) Predicting deleterious amino acid substitutions. *Genome Res.*, 11, 863–874. 11337480 10.1101/gr.176601 PMC311071

[31] Dong C., Wei P., Jian X. et al. (2015) Comparison and integration of deleteriousness prediction methods for nonsynonymous SNVs in whole exome sequencing studies. *Hum. Mol. Genet.*, 24, 2125–2137. 25552646 10.1093/hmg/ddu733 PMC4375422

[32] Schwarz J.M., Cooper D.N., Schuelke M. et al. (2014) MutationTaster2: mutation prediction for the deep-sequencing age. *Nat. Methods*, 11, 361–362. 24681721 10.1038/nmeth.2890

[33] Sun J., Jia P., Fanous A.H. et al. (2009) A multi-dimensional evidence-based candidate gene prioritization approach for complex diseases–schizophrenia as a case. *Bioinformatics*, 25, 2595–6602. 19602527 10.1093/bioinformatics/btp428 PMC2752609

[34] Muhle R., Trentacoste S.V. and Rapin I. (2004) The genetics of autism. *Pediatrics*, 113, e472–e486. 15121991 10.1542/peds.113.5.e472

[35] Klauck S.M. (2006) Genetics of autism spectrum disorder. *Eur. J. Hum. Genet*., 14, 714–720. 16721407 10.1038/sj.ejhg.5201610

[36] Freitag C.M. (2007) The genetics of autistic disorders and its clinical relevance: a review of the literature. *Mol. Psychiatry*, 12, 2–22. 17033636 10.1038/sj.mp.4001896

[37] Abrahams B.S. and Geschwind D.H. (2008) Advances in autism genetics: on the threshold of a new neurobiology. *Nat. Rev. Genet.*, 9, 341–355. 18414403 10.1038/nrg2346 PMC2756414

[38] Losh M., Sullivan P.F., Trembath D. et al. (2008) Current developments in the genetics of autism: from phenome to genome. *J. Neuropathol. Exp. Neurol.*, 67, 829–837. 18716561 10.1097/NEN.0b013e318184482d PMC2649757

[39] State M.W. (2010) The genetics of child psychiatric disorders: focus on autism and Tourette syndrome. *Neuron*, 68, 254–269. 20955933 10.1016/j.neuron.2010.10.004 PMC3292208

[40] SPARK Consortium (2018) SPARK: a US cohort of 50,000 families to accelerate autism research. *Neuron*, 97, 488–493. 29420931 10.1016/j.neuron.2018.01.015 PMC7444276

[41] Xie C., Mao X., Huang J. et al. (2011) KOBAS 2.0: a web server for annotation and identification of enriched pathways and diseases. *Nucleic Acids Res.*, 39, W316–W322. 21715386 10.1093/nar/gkr483 PMC3125809

[42] Sauter S., Beust G. von, Burfeind P. et al. (2003) Autistic disorder and chromosomal mosaicism 46,XY[123]/46,XY,del(20)(pter →p12.2)[10]. *Am. J. Med. Genet. A.*, 120A, 533–536. 12884434 10.1002/ajmg.a.20089

[43] Papanikolaou K., Paliokosta E., Gyftodimou J. et al. (2006) A case of partial trisomy of chromosome 8p associated with autism. *J. Autism Dev. Disord.*, 36, 705–709. 16602035 10.1007/s10803-006-0104-3

[44] Yurov Y.B., Vorsanova S.G., Iourov I.Y. et al. (2007) Unexplained autism is frequently associated with low-level mosaic aneuploidy. *J. Med. Genet.*, 44, 521–525. 17483303 10.1136/jmg.2007.049312 PMC2597925

[45] Castermans D., Thienpont B., Volders K. et al. (2008) Position effect leading to haploinsufficiency in a mosaic ring chromosome 14 in a boy with autism. *Eur. J. Hum. Genet.*, 16, 1187–1192. 18414512 10.1038/ejhg.2008.71

[46] Meyer K.J., Axelsen M.S., Sheffield V.C. et al. (2012) Germline mosaic transmission of a novel duplication of PXDN and MYT1L to two male half-siblings with autism. *Psychiatr. Genet.*, 22, 137–140. 22157634 10.1097/YPG.0b013e32834dc3f5 PMC3309069

[47] Krupp D.R., Barnard R.A., Duffourd Y. et al. (2017) Exonic mosaic mutations contribute risk for autism spectrum disorder. *Am. J. Hum. Genet.*, 101, 369–390. 28867142 10.1016/j.ajhg.2017.07.016 PMC5590950

[48] He X., Sanders S.J., Liu L. et al. (2013) Integrated model of de novo and inherited genetic variants yields greater power to identify risk genes. *PLoS Genet.*, 9, e1003671. 23966865 10.1371/journal.pgen.1003671 PMC3744441

[49] Willsey A.J. and State M.W. (2015) Autism spectrum disorders: from genes to neurobiology. *Curr. Opin. Neurobiol.*, 30, 92–99. 25464374 10.1016/j.conb.2014.10.015 PMC4586254

[50] Köhler S., Vasilevsky N.A., Engelstad M. et al. (2017) The human phenotype ontology in 2017. *Nucleic Acids Res.*, 45, D865–D876. 27899602 10.1093/nar/gkw1039 PMC5210535

