# Supplementary figures and images for: AutismKB 2.0: a knowledgebase for the genetic evidence of autism spectrum disorder
Source: Database (Oxford). 2018 Oct 18;2018:bay106. doi: 10.1093/database/bay106 (PMC6193446; doi:10.1093/database/bay106)

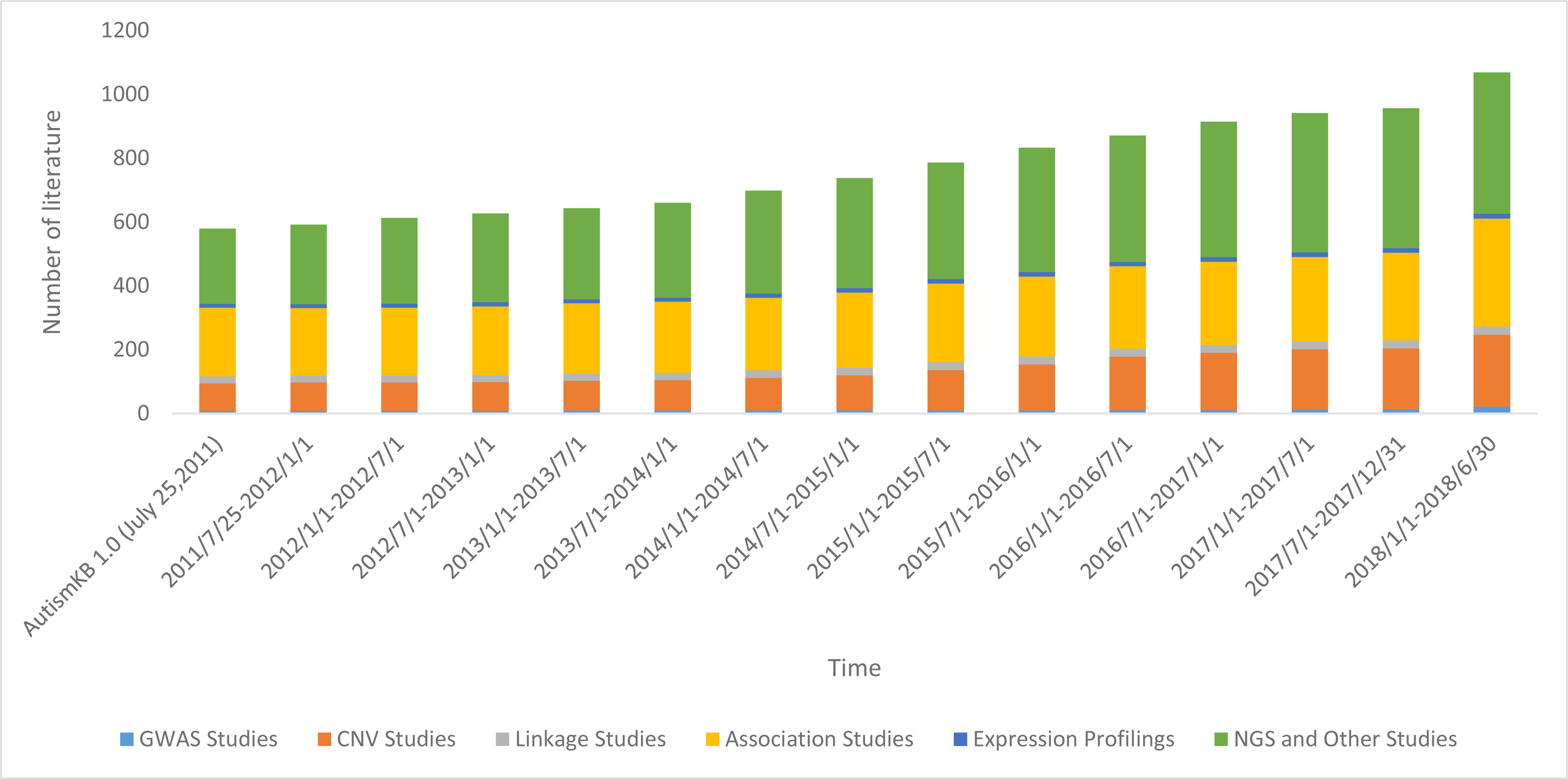

Supplement: Supplementary Data [file bay106_supp.zip › Supplementary Figure 1.jpg]
